# Transoral thyroidectomy: A reflexive opinion on the technique

**DOI:** 10.20945/2359-3997000000383

**Published:** 2021-06-29

**Authors:** Alfio Tincani, Carlos Lehn, Cláudio Cernea, Emilson Queiroz, Fernando Dias, Fernando Walder, Flávio Hojaij, Francisco Monteiro, Jacob Kligerman, José Podesta, Lenine Brandão, Luiz Eduardo Barbalho de Mello, Luiz Medina, Márcio Abrahao, Marcos Tavares, Mauro Barbosa, Onivaldo Cervantes, Paula Demétrio, Ricardo Curioso, Roberto Lima, Sérgio Arap, Sylvio Vasconcellos

**Affiliations:** 1 Universidade Estadual de Campinas Campinas SP Brasil Universidade Estadual de Campinas, Campinas, SP, Brasil; 2 Hospital do Servidor Público Estadual de São Paulo São Paulo SP Brasil Hospital do Servidor Público Estadual de São Paulo, São Paulo, SP, Brasil; 3 Faculdade de Medicina da Universidade de São Paulo Departamento de Cirurgia São Paulo SP Brasil Departamento de Cirurgia da Faculdade de Medicina da Universidade de São Paulo, São Paulo, SP, Brasil; 4 Instituto Nacional de Câncer Rio de Janeiro RJ Brasil Instituto Nacional de Câncer, Rio de Janeiro, RJ, Brasil; 5 Universidade Federal de São Paulo Escola Paulista de Medicina São Paulo SP Brasil Universidade Federal de São Paulo, Escola Paulista de Medicina, São Paulo, SP, Brasil; 6 Faculdade de Medicina da Universidade de São Paulo Laboratório de Investigação Médica Departamento de Cirurgia São Paulo SP Brasil Departamento de Cirurgia, Laboratório de Investigação Médica (LIM-02), Faculdade de Medicina da Universidade de São Paulo, São Paulo, SP, Brasil; 7 Faculdade de Medicina da Universidade de São Paulo São Paulo SP Brasil Faculdade de Medicina da Universidade de São Paulo, São Paulo, SP, Brasil; 8 Universidade Federal do Rio Grande do Norte Centro de Ciências da Saúde Programa de Pós-graduação em Ciências da Saúde Natal RN Brasil Universidade Federal do Rio Grande do Norte, Centro de Ciências da Saúde, Programa de Pós-graduação em Ciências da Saúde, Natal, RN, Brasil

## DEAR EDITORS AND COLLEAGUES

When analyzing the literature published on thyroidectomy using a “timeline” we can observe that we have notoriously progressed from a procedure that was almost prohibited in the mid-nineteenth century due to complications to a point where it is considered safe, resolutive, and highly efficient. This progress is especially due to the advances in surgical techniques. Theodore Kocher (Nobel Prize in 1909 for his contribution to thyroidectomies) flawlessly described the anatomical basis for the success of this surgical technique 130 years ago. Besides this, advances in anesthetic procedures, surgical materials, and medications were fundamental to intraoperative and postoperative advances and patient management. With time, the knowledge on the different thyroid diseases has exponentially increased and serves as indicators for the extension of treatment and surgical procedures, all efforts should be directed to apply them into clinical practice (1).

In the past twenty years, the introduction of new technologies such as the application of energy in surgical instruments and neuromonitoring brought advances to the procedure. These technologies helped providers to achieve a shorter surgical time, a shorter length of hospital stay, a reduction in the risk of bilateral laryngeal paralysis, and a reduced risk of intra and post-operative bleeding (2,3). In the last decade, stimulated by the current advances in endoscopic and robotic surgery, new approaches for thyroidectomy procedures were investigated to substitute the classic cervical incision, and are currently a theme for several scientific debates.

Previously described techniques using endoscopic and small incisions through the chest wall were proven not to be efficient and were soon taken out of practice. The same happened with the robotic techniques using the axillary route. It is worth mentioning that most of the robotic apparatus was designed for abdominal and thoracic cavity surgeries. To date, no specific instrument in the market was designed specifically for Head and Neck Surgery.

However, new technique proposals for thyroidectomy using remote access via cervicofacial routes have recently emerged. The most used pathways for access are the retro-auricular route (an incision is made at the hairline and access granted through a subcutaneous tunnel) and the transoral routes (TOETVA – where the surgical instruments are adapted from the ones used on abdominal endoscopic surgery – laparoscopies, and TOETRA – using the same robotic instruments mentioned above) (4-6).

In Brazil, about ten percent of the head and neck surgeons (out of about a thousand) are enthusiastic about the transoral technique, advocating for it to be included as a standard of care treatment in the surgical treatment of the thyroid gland (7).

In this letter, a group composed of Brazilian Head and Neck Surgery specialists is bringing their opinion in including the technique as a standard of care procedure. We will bring some points and concerns that are should be highlighted and the doubts about having these procedures (TOETVA or TOETRA) considered routine standard of care.

Publications advocating TOETVA (or TOETRA) present clear data of feasibility and remarkable aesthetic results. Some publications demonstrate similar (incipient) oncological results when compared to the classic procedure (7-10).

However, the literature fails to report some data that our group believes is of utmost importance when admitting the procedure as a possible standard of care technique. The literature lacks data on time spent on the operation, the need for conversion to the classic route, presence of bleeding, rate of infection, complications due to the route used for thyroid resection such as injury to mental nerves, and aesthetic complications such as tissue fibrosis due to the extensive dissection and inadvertent opening of the soft tissues on the anterior cervical region. Furthermore, we believe that the introduction will play an important impact on surgical costs and that there are ethical issues in terms of equity (public system and private system).

## DISCUSSION

### Limitations

It is important to highlight that one of the main limitations of endoscopic thyroidectomy is that the operative field is visualized in 2 dimensions. Furthermore, it uses rigid instruments (which mainly produce linear movements, lacking the “filtering” characteristics from the hands of surgeons (3). In this context, we should also consider the difficulties of dissecting vital structures or tissues close to those noble structures in a confined space as the endoscopic thyroidectomy narrows the visualization of the operative field. For example, there are no literature reports on the preservation of the external branches of the superior laryngeal nerve, a crucial technical aspect when operating patients that are voice professionals.

### Learning curve and surgery duration

The literature reports that a surgeon must perform around 15 TOETVA cases as a learning curve (7). In addition, during the training, operative time can be two or three times longer when compared to the classic thyroidectomy and remains long even post-learning curve. Could this curve be ethically justified in daily practices? Wouldn’t it be more appropriate to place the procedure under a research protocol? It is important to emphasize that high morbidity and unusual complications after conversion to a classic thyroidectomy have been reported (9). Therefore, when would it be appropriate to convert the procedure into classic access – acting time after a complication has been identified?

### Bleeding

Almost no major bleeding has been reported in the published case series of TOETVA and TOETRA, neither intra nor postoperative. It is noteworthy that every thyroid surgeon has already experienced the serious adverse event of having to reopen, as an emergency, the conventional cervicotomy to evacuate a hematoma and alleviate the patient’s respiratory failure. A cervical hematoma is a serious and worrisome complication. Although it is a common complication of thyroidectomy (expected in 1% of thyroidectomies), there are no reports of this occurrence in the published series of TOETVA and TOETRA. How would those cases be managed since there is no cervical incision to be opened? Wouldn’t a cervical incision with local anesthesia add more morbidity to an event that is already serious?

### Infections

There are few reported cases of infections in classic thyroidectomies. The same remains true in the published series of TOETVAS and TOETRA. However, although infection is an occasional risk in classical surgery, in TOETVA it becomes an assumed risk once the thyroid is accessed through the oral cavity, which is considered a contaminated or potentially contaminated territory (7-10).

### New complications

Entering the cervical area through unusual “portals” places risk to several anatomical structures (such as lips, mental nerves, mimic muscles, thyrohyoid membrane, and others) that are not manipulated during a conventional thyroid incision. Corroborating this statement, the literature reports complications such as labial paresthesia, due to mental nerve injury. In addition, other complications never described in conventional thyroidectomies, have been reported such as changes in smell and taste caused by using antiseptics in the areas accessed and by surgical positioning.

A new type of complication should also be highlighted: The quality of the surgical specimen. Although small incisions allow instruments to be introduced and used for dissection, they do not permit, in a considerable number of cases, the removal of the entire surgical specimen without imposing damage to the tissue. Tissue quality is of utmost importance for the histopathological assessment of lesions that are crucial for future decisions on adjuvant therapies.

It is also important to mention that TOETVAS and TOETRA imposes a risk of CO2 embolism, once it is used for tissue insufflation to maintain the operative cavity distended and allow for the visualization of anatomical structures. This new complication is not mentioned in the literature, although the theoretical risk of it happening should be more frequent than what is currently seen in abdominal and thoracic surgeries, as the neck does not have a real anatomical cavity. To insufflate gas into the cervical region, a territory of many important vessels, greater pressure is needed to create the necessary space for the operation. In addition, the literature reports a few cases of subcutaneous emphysema in patients submitted to endoscopic thyroidectomy through lateral incisions, a technique that has now been abandoned. In those cases, insufflation caused not only emphysema of the neck but also of the face and chest, with indisputable aesthetic impairments, in addition to severe pain.

### Esthetical complications

It sounds strange to discuss esthetics once TOETVA and TOETRA are techniques that propose superior esthetic results for the patients. However, it should be mentioned that there are reports of severe skin lesions happening in the surgical instrument’s pathway on aesthetically significant areas of the face and neck. Punctures and burns can present significant and unacceptable esthetical complications. For example, if the damage is inflicted on the lower lip, either sensory or motor, wouldn’t it be more visible and esthetically unpleasant than the presence of a scar from a classic neck incision? Undoubtedly, this requires consideration. Moreover, fibrosis-related skin and deep tissue retraction is an esthetical complication that has not been described in the literature, probably due to the longer time needed to occur post-operatively. Tissue fibrosis can also present with functional impairments such as dysphagia and movement discomfort. Besides this, the presence of undulations and irregularities in the skin of the cervical region, in addition to the sensation of “neck tightness” and “burning”, can also be caused by a larger dissection area needed for surgical access.

### Financial and social burdens

It is important to assess the financial and social costs of routinely considering TOETVA and TOETRA on the standard of care practices. Although not measured in the literature, there is a clear increase in real costs. Exact quantification of the increase in costs is difficult, especially when considering the subjectivity of the data. Nevertheless, qualifying is not. The procedure requires investments in surgical training, it has a longer execution time, requires a larger amount of medications to be given to the patient (for example, antibiotics are given for at least 7 days (4)), and require the use of expensive tools such as endoscopic materials. Those materials are considerably more expensive even when permanent tools are used instead of disposable ones.

The social burden of routinely offering TOETVA and TOETRA as the standard of care treatment for thyroid surgical pathologies cannot be ignored, especially when considering the reality of our country. In the Public Health System (SUS), several patients are waiting in large queues for an opportunity to be surgically treated. It does not seem appropriate to routinely offer a procedure that takes at least twice as long to be completed, as it will increase the time those patients will be waiting for treatment.

Besides this, routinely performing TOETVA and mainly TOETRA, would highly impact both the public and private health care systems due to the significant cost increase of the procedure. It would certainly impose an undesirable revision in the actuarial calculations of thyroidectomy costs. This would be particularly undesired in the difficult times in which we live now.

Finally, we would like to highlight that the literature also states the TOETVA and TOETRA techniques cannot be considered for all patients. The exclusion criteria include thyroid gland with high volume, tumor staging, presence of previous treatments, and patient anthropometrics (7-10). The defenders of the procedure consider TOETVA and TOETRA to be minimally invasive. However, transoral access should not be considered minimally invasive as they require larger tissue dissection, and access through potentially contaminated areas. Although it does not present with a visible neck incision, in our opinion, those procedures are even more invasive than the classic thyroidectomy.

## CONCLUSION

In our opinion, there is only one gain from the TOETVA and TOETRA approach which is the aesthetical appearance of the neck. We also believe that this benefit should be considered limited due to the risk of complications motioned above. Moreover, it is important to emphasize that classic thyroidectomy incisions are almost always unnoticeable and have little impact on quality of life (11,12). We strongly believe that further discussions on the risk versus benefits of those procedures should be clearly and honestly described in professional forums. We also vehemently repudiate the use of media vehicles, outside the scope of ethical and scientific discussions, for the dissemination of techniques that, in our point of view, still lack a solid foundation for routine adoption. In our opinion, as many questions remain to be answered, those procedures should stand as an exception and should be further studied before entering the routine standard of care.

In conclusion, TOETVA and TOETRA are procedures that add several unprecedented complications to thyroidectomies, take longer to perform (approximately two to three times when compared to conventional thyroidectomy), and adds costs. There is a large learning curve and it is inadequate to be carried out outside of an educational institution, as it requires experienced mentorship. The aesthetical gain of this procedure makes little sense given the excellent results obtained with the traditional procedures. The technique, mistakenly entitled “minimally invasive”, lacks the advantages widely found in video-laparoscopic surgeries. TOETVA and TOETRA are not offered as a surgical option at the main cancer treatment centers in the USA (Memorial SK Cancer Center and MD Anderson) nor in the guidelines of the American Society of Endocrine Surgeons (ATA). We strongly believe that those procedures should be considered experimental until further data is available to ensure patient safety.
